# Scale-Up Studies for Co/Ni Separations in Intensified Reactors

**DOI:** 10.3390/mi11121106

**Published:** 2020-12-15

**Authors:** Dimitrios Tsaoulidis, Milan Mamtora, Marta Mayals Gañet, Eduardo Garciadiego-Ortega, Panagiota Angeli

**Affiliations:** 1 Chemical and Process Engineering Department, University of Surrey, Guildford GU2 7XH, UK; 2 ThAMeS Multiphase, Chemical Engineering Department, UCL, London WC1E 7JE, UK; milan.mamtora.15@alumni.ucl.ac.uk (M.M.); marta.ganet.15@alumni.ucl.ac.uk (M.M.G.); e.garciadiego-ortega@ucl.ac.uk (E.G.-O.)

**Keywords:** multi-component extraction, scale-up, Co/Ni separations, process intensification, microchannels, liquid–liquid plug flow

## Abstract

In this paper, the effect of the scalability of small-scale devices on the separation of Co(II) from a binary Co(II)/Ni(II) mixture in a nitric acid solution by an organic Cyanex 272/TBP/kerosene (Exxsol D80) phase is studied. In particular, circular channels with diameters of 1, 2, and 3.2 mm are considered. The results were compared against those from a confined impinging-jets (CIJ) cell with a main channel diameter of 3.2 mm. The effects of total flowrate, residence time, Cyanex 272 concentration, and flowrate ratio on the mass transfer performance were investigated. It was found that at increased channel size, the throughputs were also increased but the extraction percentages remained the same. Higher extraction percentages were obtained by using the CIJ configuration at short residence times. However, for longer residence times, the mass transfer coefficients were similar and capillary channels should be preferred over the CIJ because of the ease of separation of the two phases at the end of the unit. The overall mass transfer coefficients ranged between 0.02 and 0.14 s^−1^ for the capillary channels during plug flow and between 0.05 and 0.45 s^−1^ for the CIJ cells during dispersed flow.

## 1. Introduction

Metals are central elements in everyday life; many have experienced an increase in demand in recent years due to their widespread use in industrial processes and consumer goods [1]. Cobalt and nickel, in particular, are among the most important non-ferrous metals, and they mostly appear together, making their separation a key process in hydrometallurgy [2]. Their superior strength under extreme temperatures, ductility, and corrosion resistance make them valuable materials for the manufacture of stainless steel and alloys, with uses in the chemical, petrochemical, and power generation industries. They are generally retrieved from sulphide and laterite ores, which are leached in acid solutions to give a pregnant leach solution that contains soluble salts of cobalt, nickel, iron, magnesium, and, at lower concentrations, chromium, aluminium, and manganese [3]. Once the impurities are removed via precipitation, final separation of Co and Ni is required. Cobalt and nickel are transition metals with similar physicochemical properties, and they cannot be easily separated via simple methods such as chemical precipitation by pH adjustment when they are both present in aqueous solutions [4]. The separation and recovery of Co from Ni has mainly been conducted through direct solvent extraction from aqueous media (sulphuric, hydrochloric, or nitric acid solutions) by employing different types of extractants [5,6]. Cobalt is selectively removed from the aqueous solution by reacting with the extractant and replacing the hydrogen ions in the latter, thus forming an organometallic complex. Among the many extractants reported for Co(II)/Ni(II) separation, phosphorus-based extractants such as di-2-ethylhexyl phosphoric acid (D2EHPA), 2-ethylhexyl phosphonic acid mono-2-ethylhexyl ester (P507 or PC88A), and bis (2,4,4-trimethylpentyl) phosphinic acid (Cyanex 272) have shown superior extraction performance in acidic solutions [7]. Cyanex 272 gives extraction efficiency and can separate cobalt from both low and high Ni content solutions, but it has not been adopted widely by industry due to its high cost. Combined with the use of conventional extraction equipment such as mixer-settlers and pulsed columns, characterized by large liquid inventories, long residence times (ranging from a few minutes to hours), difficulties in process control, and large footprint, this leads to increased capital and operating costs [8,9].

New intensified technologies can provide faster, cheaper, and greener processes [10]. In recent years, intensified units where two immiscible liquids are involved have attracted much attention in process engineering for the separation, purification, and recycling of precious metals [11,12], rare earth metals [13,14], and actinides [15,16,17,18]. A common intensification approach involves the use of small channels, where thin fluid films form that decrease the diffusion distances and increase the mass transfer rates. The flow patterns that mainly form during the flow of two immiscible liquids in small channels range from parallel to transitional and to plug (segmented) flows [19]. The plug flow pattern has been extensively studied for different applications. In this pattern, one phase forms dispersed droplets (plugs) whose equivalent diameter is larger than the channel diameter, whilst the other phase is continuous (slug) and surrounds the plugs separating them from each other as well as from the channel wall via a thin film. During plug flow, circulation patterns establish within each phase and improve radial mixing whilst, due to the very thin film next to the wall, axial mixing is limited [20]. For industrial applications, however, it is important to increase the throughput that can be processed. To increase throughput, increases in channel sizes should be considered that still preserve the benefits of small-scale operations, such as thin fluid films and enhanced mass transfer rates. An alternative option for increasing throughput is the confined impinging-jets (CIJ) inlet configuration [21,22]. In this case, the two immiscible liquids are brought into contact in the inlet at high velocities, increasing significantly the energy dissipation rates at this region [23]. With this configuration, dispersed flow is formed, which exhibits very large specific interfacial areas available for mass transfer.

Over the last decade, small-scale devices have been applied to the Co/Ni separations, and both the hydrodynamics and mass transfer have been investigated. Previous studies are summarized in Table 1. It was found that high extraction efficiencies can be obtained in capillary channels at short residence times, whilst the values of overall mass transfer coefficients (kLα) achieved were one to two orders of magnitude higher than in conventional equipment [6,24,25]. Furthermore, it has been possible to derive reliable kinetics of the separations of multicomponent mixtures using Cyanex 272, because of the fast mixing in the microchannels, which can help to optimize the reactor design [26].

For industrial applications, it is important to increase the throughput of small-scale devices. The effect of channel size, however, on the extraction performance of the intensified flow separators has not been studied previously. In this paper, the effect of channel size on the continuous separation of Co(II) ions from a binary mixture of Co/Ni in acidic solutions is studied, using Cyanex 272 as the extractant. The effects of different parameters such as total flowrate, flowrate ratio, residence time, and extractant (Cyanex 272) concentration are considered. Mass transfer coefficients are compared against those from confined impinging-jets (CIJ) cells for the same extraction system.

## 2. Materials and Methods

### 2.1. Materials

Cobalt(II) nitrate hexahydrate Co(NO_3_)_2_·6H_2_O (purchased from Sigma-Aldrich, Darmstadt, Germany; purity > 99%) 6 mg/mL and nickel(II) nitrate hexahydrate Ni(NO_3_)_2_·6H_2_O (purchased from Sigma-Aldrich; purity > 99%) 60 mg/mL were dissolved in nitric acid solutions (HNO_3_ 3 M) to form the aqueous phase. The organic phase was prepared by mixing kerosene (Exxsol D80, purchased from Brenntag, Essen, Germany), tributylphosphate [TBP; O=P(OC_4_H_9_)_3_] (purchased from Sigma Aldrich; 97% purity) as modifier at constant volume concentration (5% *v*/*v*) and the phosphinic acid Cyanex 272 (purchased from Sigma-Aldrich; purity 90%) as the extractant at different volume concentrations (10–30% *v*/*v*). Sodium hydroxide (purchased from Sigma-Aldrich; 98% purity) was used for the pre-neutralization of the organic phase.

### 2.2. Experimental Setup and Procedure

**Figure 1:**
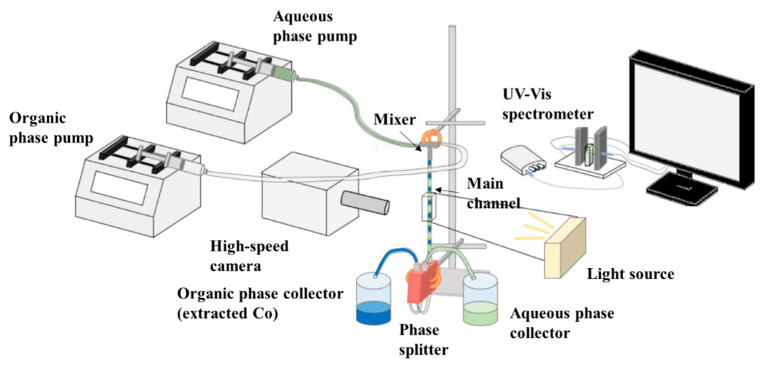
Schematic of the experimental setup for the continuous Co/Ni separation.

**Figure 2:**
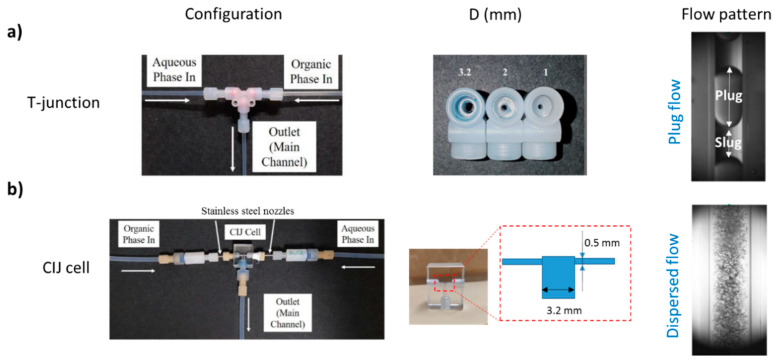
Images of the small-scale inlets used and indicative photos of flow patterns obtained in (**a**) T-junction (plug flow) and (**b**) Confined impinging-jets (CIJ) cell (dispersed flow).

The continuous separation in flow channels of cobalt from a cobalt/nickel mixture in a nitric acid solution by the organic phase (D80/TBP/Cyanex272) was performed in the experimental setup shown in Figure 1. Two high-precision pumps were used to feed the organic and aqueous phases independently into the main separation channel via a T-junction. The T-junction was made of FEP (fluorinated ethylene propylene) with the side branches having the same diameter (D = 1, 2, or 3.2 mm) as the main channel, as shown in Figure 2a. In all cases, the effect of residence time was investigated by adjusting the length of the main channel, while the flowrates of the two phases remained the same to ensure that the flow pattern and the mixing characteristics did not change. The results were compared with initial findings from a confined impinging-jets (CIJ) cell. The CIJ was made of acrylic using a CNC machine. Two stainless steel nozzles with diameter, d_n,_ of 0.5 mm, were inserted opposite to each other and perpendicular to the main channel that had 3.2 mm diameter (D), as shown in Figure 2b.

At the end of the main channel, a phase splitter (SEP-10; purchased from Zaiput Ltd., Boston, MA, USA) was used to fully separate the two phases. For the separation, a hydrophobic membrane of medium pore size was used (purchased from Zaiput Ltd.). After the separation, a UV-Vis spectrometer was used to measure the absorbance of the Co(II) and Ni(II) ions in the aqueous phase, at 510 and 720 nm, respectively, for the calculation of the concentration. This method resulted in an average error of 4.5% for the estimation of the extraction percentage. The hydrodynamic features during the two-phase flow were captured using a CMOS high-speed camera (V1212 Phantom, Vision Research, Wayne, NJ, USA) with maximum resolution of 1280 × 8000 pixels operated at 12,600 fps, equipped with a magnification lens (×12, LEICA Monozoon 7 optical system, Wetzla, Germany). The pixel size varied from 3.2 to 9.5 μm. It was found that a very regular plug flow pattern was established and the plug and slug lengths, averaged over 40–70 images, had a standard deviation below 2%. The physical properties of the two phases were measured experimentally and are shown in Table 2. The viscosity of the fluids was measured in a Modular Compact Rheometer MCR 302 (purchased from Anton Paar, Graz, Austria), the density was obtained with a density bottle (Sigma-Aldrich), and the surface and interfacial tension were measured using a Kruss Tensiometer DSA100 (Hamburg, Germany).

To calculate the distribution coefficients of the binary mixture Co/Ni, batch equilibrium separation experiments were performed. The effects of extractant concentration, pre-neutralization level, and equilibrium pH_eq_ were evaluated. For these studies, the two phases were mixed at appropriate volumes (depending on the phase ratio used in the flow experiments) in a beaker with a mechanical stirrer until equilibrium was reached (0.5 h).

For the continuous flow separation of Co(II) ions from the binary mixture Co/Ni, capillary channels with diameters (D) of 1, 2, and 3.2 mm were used with the T-junction inlet. The effects of different independent variables, as shown in Table 3, on extraction performance were analyzed during plug flow. In the confined impinging-jets cell, different total flowrates (and residence times) were used, whilst the phase flowrate ratio and the Cyanex 272 concentration were constant at 1 and 20% *v*/*v*, respectively. In all the experiments, the equilibrium pH was set to 4.5.

To evaluate the mass transfer performance, the following descriptors were calculated, i.e., extraction percentage (E%), distribution coefficient (K_i_), separation factor (β_Co/Ni_), and mass transfer coefficient (k_L_α):

(1) $$E_{i}=\frac{C_{\mathrm{aq},\mathrm{init},i}-C_{\mathrm{aq},\mathrm{fin},i}}{C_{\mathrm{aq},\mathrm{init},i}}$$

(2) $$K_{i}=\frac{C_{\mathrm{org},\mathrm{eq},\;i}}{C_{\mathrm{aq},\mathrm{eq},i}}$$

(3) $$\beta_{\mathrm{Co}/\mathrm{Ni}}=\frac{K_{\mathrm{Co}}}{K_{\mathrm{Ni}}}$$

(4) $$k_{L}\alpha =\frac{1}{t}\ln\left(\frac{C_{\mathrm{aq},\mathrm{eq},i}-C_{\mathrm{aq},t_{\mathrm{fin}},i}}{C_{\mathrm{aq},\mathrm{eq},i}-C_{\mathrm{aq},t_{\mathrm{init}},i}}\right)$$

where i = Co or Ni, $C_{\mathrm{aq},\mathrm{init}}$ is the concentration of a species in the aqueous phase initially, $C_{\mathrm{aq},\mathrm{fin}}$ is the concentration of a species in the aqueous phase at the outlet, $C_{\mathrm{aq},\mathrm{eq}}$ is the concentration of a species in the aqueous phase at equilibrium, and $C_{\mathrm{org},\mathrm{eq}}$ is the concentration of a species in the organic phase at equilibrium.

## 3. Results and Discussion

### 3.1. Separation of Co from Ni/Co Mixtures at Equilibrium

The extraction of Co(II) and Ni(II) ions by Cyanex 272 can be written as shown in Equations (5) and (6), respectively.

(5) $${\mathrm{Co}}_{\mathrm{aq}}^{2+}+2H_{2}A_{2,\mathrm{org}}↔\mathrm{Co}{\left({\mathrm{HA}}_{2}\right)}_{2,\mathrm{org}}+2H_{\mathrm{aq}}^{+}$$

(6) $${\mathrm{Ni}}_{\mathrm{aq}}^{2+}+3H_{2}A_{2,\mathrm{org}}↔\mathrm{Ni}{\left({\mathrm{HA}}_{2}\right)}_{2}{\left(H_{2}A_{2}\right)}_{\mathrm{org}}+2H_{\mathrm{aq}}^{+}$$

H_2_A_2_ and HA_2_ denote the complexes of the extractant. During the separation of Co(II) ions from the Co/Ni binary mixture using Cyanex 272, protons H^+^ are exchanged with Co(II) ions, which means that equilibrium can be shifted by controlling the pH of the reaction. The Cyanex 272 extractant was pre-neutralized with sodium hydroxide (NaOH) to promote the deprotonation of Cyanex (Equations (5) and (6)) and maintain the pH at an optimum value during extraction. The neutralization reaction is given by Equation (7) [30].

(7) $${\mathrm{Na}}_{\mathrm{aq}}^{+}+1/2{\left(\mathrm{HA}\right)}_{2\mathrm{org}}\to {\mathrm{NaA}}_{\mathrm{org}}+H_{\mathrm{aq}}^{+}$$

**Figure 3:**
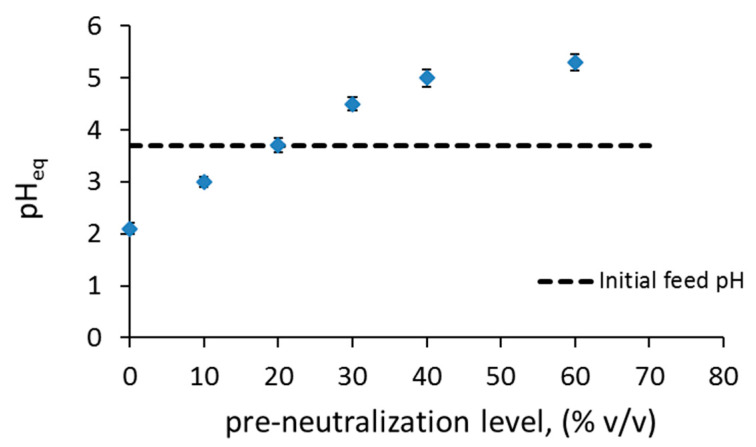
Effect of pre-neutralization level on equilibrium pH after separation of Co and Ni, using 20% *v/v* Cyanex 272, 5% *v/v* TBP, 75% *v/v* kerosene.

**Figure 4:**
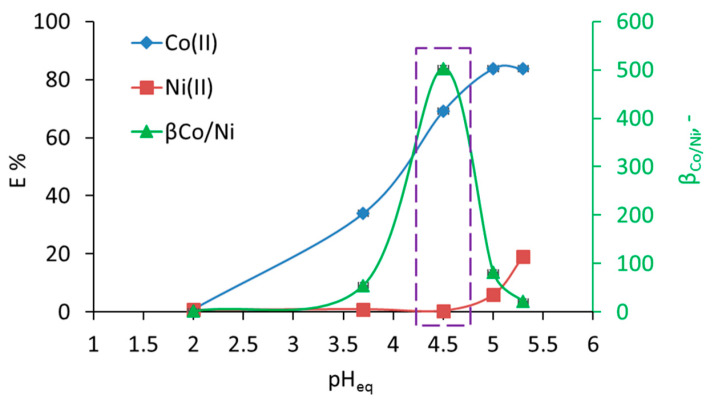
Effect of equilibrium pH (pH_eq_) on the extraction percentage of Co(II) and Ni(II), and separation factor between Co(II) and Ni(II), at 20% *v/v* Cyanex 272, 5% *v/v* TBP, 75% *v/v* kerosene (Exxsol D80).

**Figure 5:**
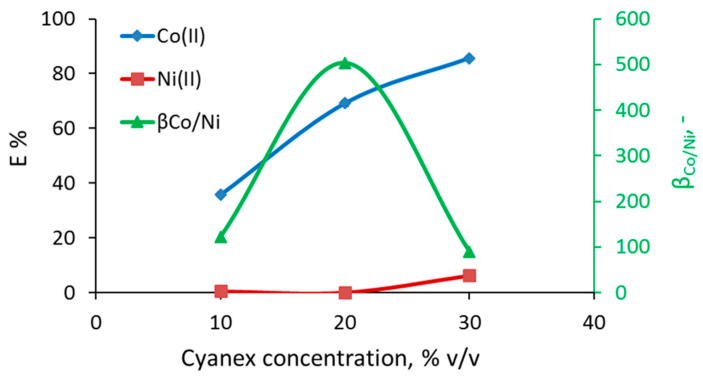
Effect of Cyanex 272 concentration (% *v*/*v*) on the extraction percentage of Co(II) and Ni(II), and separation factor between Co(II) and Ni(II), at pH_eq_ = 4.5 (30% pre-neutralization).

Therefore, initial equilibrium batch experiments were performed to identify the optimum conditions for efficient separations, since many variables can affect this multi-component system. The effect of equilibrium pH, pre-neutralization level with NaOH, and Cyanex 272 concentration on the extraction percentage and separation factor were investigated and are shown in Figure 3, Figure 4 and Figure 5.

In Figure 3, the effect of different pre-neutralization levels (*v*/*v* % of NaOH used) on the equilibrium pH (pH_eq_) is shown. The equilibrium pH increases linearly as the level of neutralization increases for values up to 40%, whilst further increase does not change the pH_eq_ significantly. It can be seen that for the initial nitric acid feed solution of 3.7% *v/v* with metals, the pH_eq_ reduces to 2.1 when no neutralization takes place, because of the release of protons H^+^.

From Figure 4, it can be noted that as the pH_eq_ increases, the extraction of Co(II) also increases; the steep increase for pH_eq_ from 2 to 5 shows how sensitive the extraction performance is to pH_eq_, with the extraction percentage varying from almost 0 to 85%. The extraction of Ni(II) is not affected for pH_eq_ values up to 4.5, which corresponds to pre-neutralization level up to 30%. However, further increase in the pH_eq_, leads to co-extraction of Ni and the separation factor, β_Co/Ni,_ decreases sharply. The optimum operation range is for pH_eq_ from 4.2 to 4.7, where the separation factor varies between 350 and 500.

The effect of Cyanex 272 concentration on the separation of Co(II) from the binary mixture Co/Ni was investigated in the range 10–30% *v*/*v*. The extraction percentage of Co(II) increases almost linearly by increasing the Cyanex 272 concentration and varies between 35 and 85% (Figure 5). However, at high Cyanex concentration (30% *v*/*v*), Ni(II) was also co-extracted. The additional H_2_A_2_ molecules in the Ni complex (Equation (6)) shift the dependence of metal distribution upon extractant concentration to higher values, which results in lower separation factors as the extractant concentration increases (Figure 5). It is worth mentioning that an increase in the extractant concentration also increases the viscosity of the organic phase and therefore the pressure drop and the overall pumping requirements. Therefore, the optimum concentration for the continuous experiments was set to 20% *v*/*v*.

### 3.2. Separation during Flow—Effect of Scaling-Up

**Figure 6:**
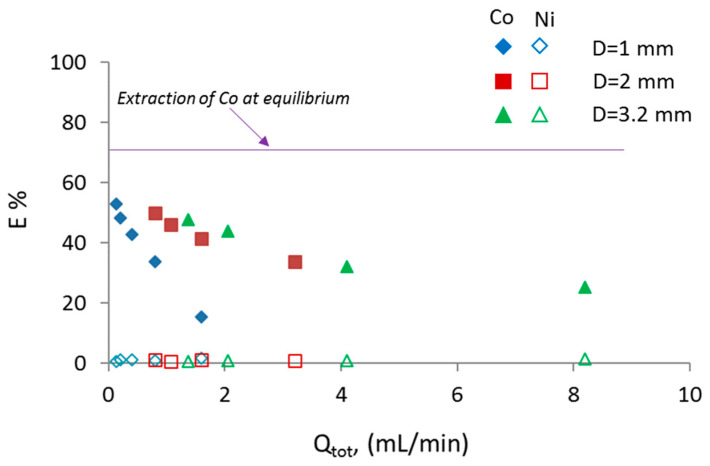
Extraction percentage of Co(II) and Ni(II) as a function of total flowrate (Q_tot_) at different channel sizes for a constant reactor length (L_r_ = 8.5 cm).

The effect of channel size on mass transfer during the flow in the channels was investigated for different operating conditions. For all conditions studied, plug flow was established in the small channels (as shown in Figure 2a). In Figure 6, the effect of total flowrate (Q_tot_) at phase flowrate ratio 1 on the % extraction at the three different channel sizes is shown. Depending on the channel size, the specific interfacial area changes; it decreases by increasing the channel size. The specific interfacial area for the different channel sizes 1, 2, and 3.2 mm ranged between 1900 and 2570, 975 and 1310, 670 and 880 m^2^/m^3^, respectively. As can be seen, with increasing channel size, the range of specific interfacial area values obtained decreases. For a certain channel size, the specific interfacial area increased by increasing the flowrate. Even though the increase in total flowrate resulted in higher specific interfacial areas due to smaller plug lengths, the plug formation time decreased, resulting in less contact time of the phases in the mixing zone. Previous studies have shown that the effect of mixing zone on the overall extraction efficiency of the reactor contributes from 25 to 40% depending mainly on the flowrate ratio, the total flowrates, and the channel size [13,16,31].

For a constant mixture velocity, the plug formation time increases by increasing the channel size. For all three sizes, it can be seen that for a constant reactor length of 8.5 cm, the extraction percentage decreases as the total flowrate increases, which is expected because the residence time decreases. The residence time varied between 2.5 and 30 s for all the cases shown in Figure 6. The decrease in extraction percentage with total flowrate is more obvious in the smaller channel (1 mm), whilst for the 2 and 3.2 mm channels, the decrease is less. For instance, for a four-fold increase in the total flowrate, i.e., from 0.2 to 0.8 mL/min (1 mm) and from 2.05 to 8.2 mL/min (3.2 mm), the extraction percentage decreases by ~53% in the case of the 1 mm channel, whilst in the case of the 3.2 mm channels, it decreases by ~37%.This trend indicates that for a certain channel length, an increase in channel size allows higher throughput without reducing the extraction percentage. For example, for a constant flowrate, i.e., 1.6 mL/min, the extraction percentage increases 2.5 times when the channel size increases from 1 to 2 or 3.2 mm. This is mainly attributed to the fact that during plug flow, a large percentage (~35%) of mass transfer takes place during plug formation in the mixing zone [13,15]. It can also be seen that Ni(II) was not co-extracted in all cases, in agreement with the equilibrium results.

**Figure 7:**
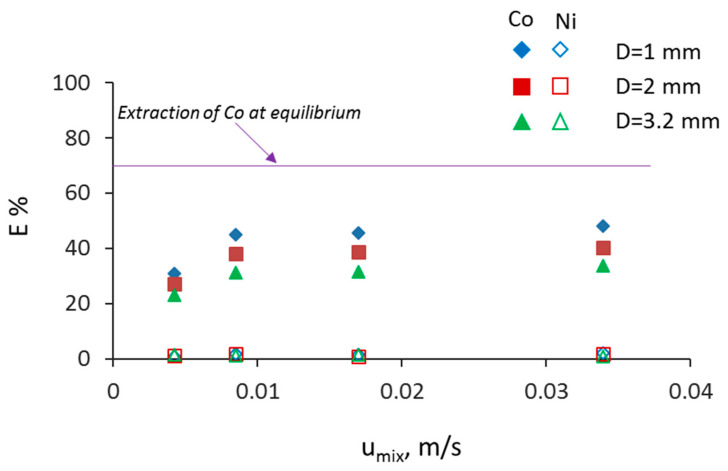
Extraction percentage of Co(II) and Ni(II) as a function of mixture velocity (Q_tot_) at different channel sizes at constant residence time (t = 10 s).

**Figure 8:**
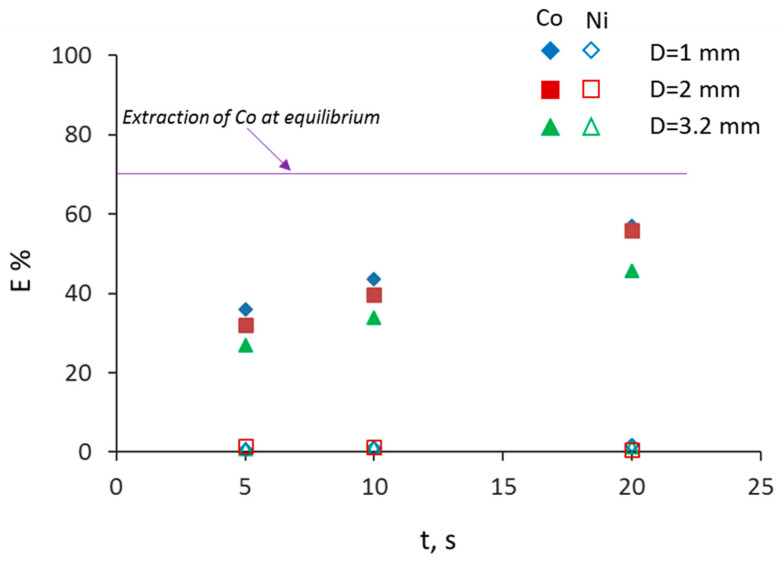
Extraction percentage of Co(II) and Ni(II) as a function of residence time (t) at different channel sizes at constant mixture velocity (u_mix_ = 0.017 m/s).

To study the effect of mixture velocity for constant residence time, different channel lengths were used. In Figure 7, the effect of mixture velocity, u_mix_, for the different channel sizes is shown for a residence time of t = 10 s. As can be seen, an increase in the mixture velocity increased initially the extraction percentage for all channel sizes. The increase is larger in the 1 mm channel compared to the other two channels and is attributed to the shorter diffusion lengths and the faster internal circulations in the plugs and the slugs [31]. At mixture velocities above 0.085 m/s, the extraction percentage reaches a plateau depending on the channel size. At the lower bound of mixture velocities (u_mix_ = 0.0042 m/s), the extraction performance is similar for all channel sizes. This is attributed to the fact that for a constant mixture velocity, the formation of the plug in the T-junction takes longer as the channel size increases, and a considerable amount of mass transfer takes place in the mixing zone [13,15]. The differences in extraction for the various channel sizes are larger than the experimental error. The effect of residence time on extraction percentage is shown in Figure 8, and as expected, by increasing the residence time, there is an increase in the extraction percentage for all channels. It can be seen that in the first 20 s, for the 1 and 2 mm channels, the extraction efficiency reached approximately 86%.

### 3.3. Effect of Cyanex 272 Concentration

**Figure 9:**
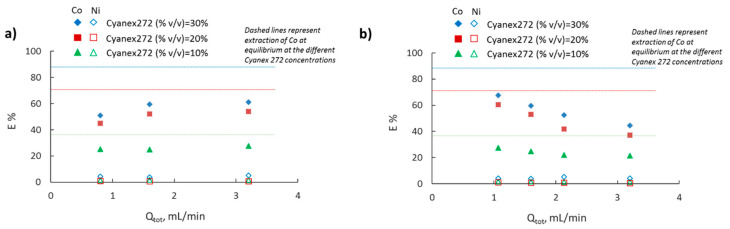
Extraction percentage of Co(II) and Ni(II) in the 2 mm channel as a function of total flowrate (Q_tot_) at different Cyanex 272 concentrations (**a**) at constant residence time (t = 20 s), and (**b**) at constant reactor length (L_r_ = 17 cm).

The amount of the extractant in the organic phase plays a crucial role both for the extraction percentage of Co(II) and the separation factor, as indicated by the equilibrium results (Figure 5). As mentioned previously, the active component of Cyanex 272 is selective for Co(II) in the presence of Ni(II), and Co ions are extracted through a cation exchange mechanism. The effect of different Cyanex 272 concentrations is shown in Figure 9. The concentration varied between 10 and 30% *v/v* and results are shown for the case of the 2 mm channel size.

In Figure 9a, the extraction percentage is plotted as a function of total flowrate for constant residence time (t = 20 s). In agreement with the equilibrium results (Figure 5), the extraction percentage increases by increasing the Cyanex 272 concentration for all operating conditions. It can be seen that for the lower Cyanex 272 concentration (10% *v*/*v*), an increase in the total flowrate has no effect on the extraction percentage. However, at the higher Cyanex 272 concentrations, 20 and 30% *v*/*v*, the extraction percentage increases with the total flowrate. At equilibrium, for an increase in the Cyanex 272 concentration from 20 to 30% *v*/*v*, the extraction percentage increases by ~24%. However, in the continuous separators, the increase in the extraction percentage is approximately ~13%. Although within the range of Cyanex 272 concentration investigated in this work, the extracted Co complexes at equilibrium have the final form of Co(HA_2_)_2_ as shown in Equation (5), the continuous experimental results might suggest that the Co migration process proceeds in the form of different complexes before the final stage is reached. Regarding the extraction of Ni, and in agreement with equilibrium results, low extraction percentage was observed (1–6%).

As can be seen in Figure 9b, for a constant reactor length, as the extractant concentration increases, there is a stronger relation between extraction percentage and total flowrate. Since the reactor length is constant (L_r_ = 17 cm), residence time decreases by increasing the total flowrate, ranging from 10 to 30 s. Comparing the 20 and 30% *v/v* Cyanex 272 concentration, one can notice that higher extraction efficiency can be achieved for the whole range of operating conditions in the case of 20% *v*/*v*, since the extraction percentage is closer to the final equilibrium values, than in the case of 30% *v*/*v*.

### 3.4. Effect of Phase Ratio

**Figure 10:**
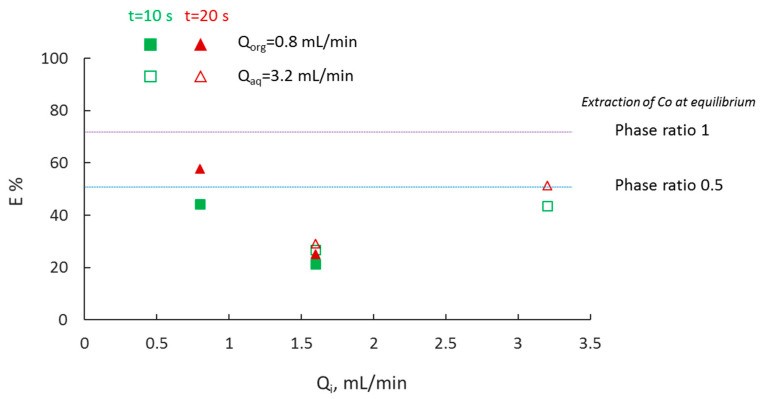
Extraction percentage of Co(II) at different volume phase ratios. (Filled symbols correspond to constant flowrate of the organic phase, whilst empty symbols correspond to constant flowrate of the aqueous phase. i denotes either org or aq.).

One of the merits of operating in small channels during plug flow is that the increased surface area to volume ratio, and the high mixing intensity due to internal circulations in the two phases, can compensate for the decreased solvent volume and can eventually lead to reduced use of solvent. The impact of phase ratio on the extraction percentage is shown in Figure 10. Cyanex 272 concentration was still in excess even for phase ratio 0.5. Results indicate that the extraction percentage decreases by decreasing the organic to aqueous phase flowrate ratio (Q_org_/Q_aq_). For a constant flow of the organic phase, Q_org_ = 0.8 mL/min (filled symbols) a decrease in the organic to aqueous phase ratio from 1 to 0.5 decreases the extraction percentage. Interestingly, at phase ratio 1, the residence time affects the results more than in the case of 0.5. For example, by increasing the residence time from 10 to 20 s, the extraction percentage increases by 30% in the case of phase ratio 1, and by 16% in the case of phase ratio 0.5. When the aqueous flow rate was kept constant, Q_aq_ = 3.2 mL/min (empty symbols), a similar trend was observed; however, the extraction percentage changes at a slower rate.

### 3.5. Overall Volumetric Mass Transfer Coefficient and Comparison of Intensified Extractors

**Figure 11:**
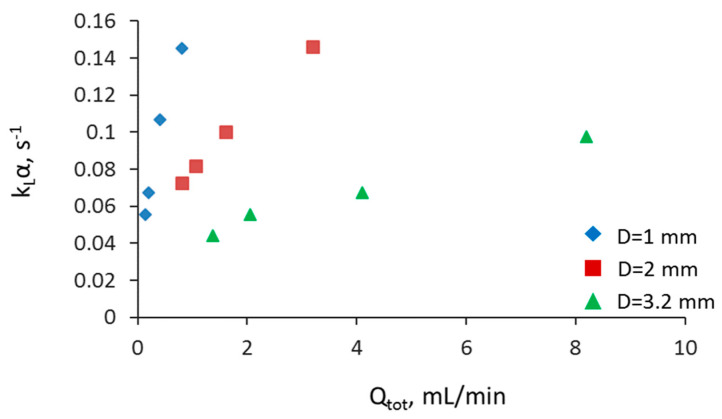
Effect of total flowrate on the overall mass transfer coefficient at different channel sizes and at constant reactor length (L_r_ = 8.5 cm).

The overall volumetric mass transfer coefficient was also evaluated to enable direct comparison of the extraction performance among different intensified separators. The mass transfer coefficients are calculated for the whole channel, including the mixing zone (T-junction), and are presented only in terms of Co(II) extraction. In Figure 11, the effect of flowrate on the mass transfer coefficient for the three capillary channels is shown. As the total flowrate increases, there is an increase in the k_L_α in all three channel sizes. Mass transfer coefficient values up to 0.14 s^−1^ can be achieved in the 1 and 2 mm channels. Results showed that the effect of flowrate on k_L_α increases as the channel size decreases. It can also be seen that for a constant reactor length, k_L_α values in the large channel comparable to those in smaller channels can be obtained by increasing the total flowrate.

**Figure 12:**
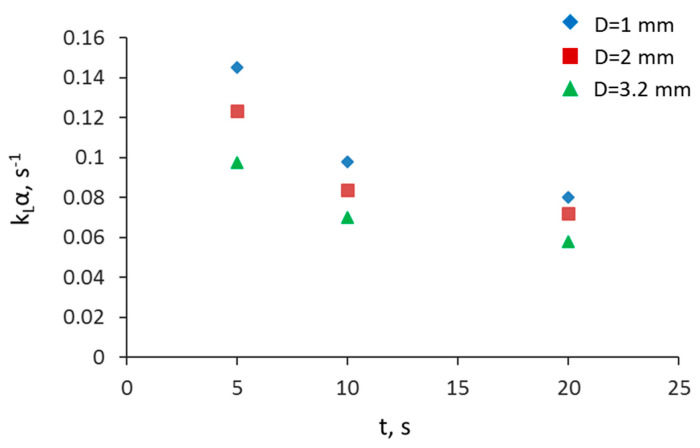
Effect of residence time on the overall mass transfer coefficient at different channel sizes and at constant mixture velocity (u_mix_ = 0.017 m/s).

In Figure 12, the overall mass transfer coefficient is plotted against the residence time at a constant mixture velocity (u_mix_ = 0.017 m/s). The k_L_α for the entire channel decreases as the channel size and the residence time increase and the values range from 0.05 to 0.14 s^−1^. Results show that for short residence times, smaller channels have high mass transfer coefficients compared to larger ones, but at long residence times, similar mass transfer coefficients are obtained in all channels. As was discussed above, higher mass transfer rates are expected in this mixing zone compared to the rest of the channel where the plug flow is fully developed [13,15]. At the inlet of the channel, though, new interfaces form and the mixing is significant as the two liquids come into contact and change flow direction. As a result, the mass transfer coefficient is expected to be high at short residence times (and short channels) where the effect of the mixing zone is significant. At longer residence times and channel lengths, the effect of the mixing zone is less important and the overall mass transfer coefficient reaches a (lower) constant value characteristic of the fully developed plug flow.

**Figure 13:**
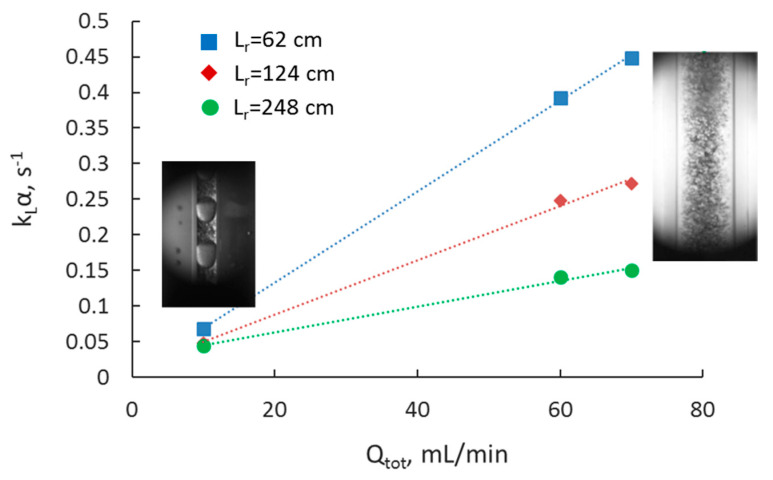
Overall volumetric mass transfer coefficient as a function of total flowrate at different reactor lengths for the CIJ cell. Photos indicate the flow pattern obtained at low and high total flow rates.

A different intensified separator (CIJ) was tested, whereby the side channels of the T-junction where smaller than the main one. The mass transfer coefficients can be seen in Figure 13 for a configuration that has a main channel with 3.2 mm diameter and side channels with 0.5 mm diameter. The large velocities in the small side channels result in high energy dissipation rates and reduce significantly the drop size formed, thus increasing the interfacial area available for mass transfer. In general, it was found that k_L_α ranged from 0.05 to 0.45 s^−1^. In Figure 13, overall mass transfer coefficients are plotted for different main channel lengths. The mass transfer coefficient increases almost linearly with total flowrate, or decreasing residence time, suggesting that there is good mass transfer at the beginning of the separator. The effect of total flowrate increases as the reactor length decreases. The higher values that were obtained for the shorter length reactor, indicate the good mass transfer that takes place in the beginning of the reactor, which includes the mixing zone.

**Figure 14:**
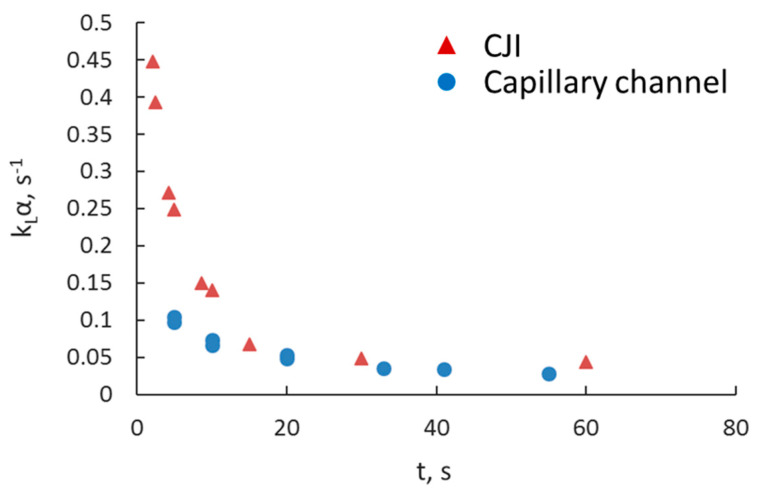
Overall volumetric mass transfer coefficient as a function of residence time for the 3.2 mm T-junction capillary channel and the 3.2 mm CIJ cell.

The mass transfer coefficients of the capillary (Q_tot_ = 1–17 mL/min) and the CIJ separators (Q_tot_ = 10–70 mL/min) with a main channel diameter of 3.2 mm are shown in Figure 14 as a function of the residence time. It can be seen that in both units, k_L_α decreases exponentially with residence time until it reaches a plateau around 0.04 s^−1^. At short residence times, higher mass transfer coefficients can be obtained in the CIJ separator compared to the channel one. However, at residence times above 20 s, the mass transfer coefficients between the two devices are similar, indicating that from an operation point of view, the capillary channels should be preferred because they allow easier separation of the two phases at the end.

## 4. Conclusions

In this paper, the separation of Co(II) from a binary mixture of Co(II)/Ni(II) in acidic solutions was investigated in small channels of various sizes to understand the effect of scale on the mass transfer performance. The effects of total flowrate, residence time, Cyanex 272 concentration, and flowrate ratio were considered. Scaling up the microreactors to process higher throughputs was possible without compromising the mass transfer efficiency. The results were compared against those from a confined impinging-jets separation unit that had a main channel of 3.2 mm diameter, similar to the largest of the capillaries used. Mass transfer coefficients varied for the capillary reactors during plug flow within the range of 0.02–0.14 s^−1^ and for the CIJ during dispersed flow within the range of 0.05–0.45 s^−1^. Overall mass transfer coefficients were two to three orders of magnitude higher than in conventional contactors. For processes of short duration, the CIJ configuration is more suitable because of the increased mass transfer that takes place with the intense mixing in the inlet. For longer processes, however, the capillary channels may be preferable because they allow easier separation of the two phases at the end of the reactor.

## Figures and Tables

**Table 1:** Summary of experimental investigations of Co/Ni separations in intensified devices.

Method and Device	Multi-Component System	k_L_α (s^−1^)	Reference
Micro-scale coiled flow inverter D = 1 mm	Co(II)/Ni(II) sulphate solution Cyanex 272	0.053–0.013	[24]
3 D conical microreactor, 12 mL internal volume	Co(II)/Ni(II) sulphate solution Cyanex 272	0.011–1.20	[25]
Flat membrane microreactor 37–74 μL volume	Co(II)/Ni(II) sulphate solution Cyanex 272	k_L_ = 2.8–5.5 × 10^−6^ m/s	[26]
Capillary microreactors D = 1 mm	Zn-Ni-Co sulphate solution D2EHPA	0.02–0.19	[27]
Counter-current flow interdigital micromixer 40 μm width channels	Co(II)/Ni(II) sulphate solution PC88A	N/A	[28]
Impinging stream-rotating packed bed contactor 1.5 mm capillary nozzles	Co(II)/Ni(II) chloride solution P507	0.16	[6]
Hollow fiber module with mixer-settler	Ionquest 801 Cyanex272	k_L_ = 4.3–7.3 × 10^−7^ m/s	[29]

**Table 2:** Properties of the aqueous and organic phases.

Properties	Aqueous Solution (Co/Ni = 6/60 mg/mL)	Organic Solution (75% *v/v* Exxsol D80, 20% *v/v* Cyanex 272, 5% *v/v* TBP)
Viscosity (kg m^−1^ s^−1^)	1.2 × 10*^−^*^3^	3.3 × 10^−3^
Surface tension (N m^−1^)	64.7 × 10*^−^*^3^	23.2 × 10^−3^
Density (kg m^−3^)	1137	831
Interfacial tension (N m^−1^)	11.1 × 10*^−^*^3^	

**Table 3:** Summary of the variables and the range investigated.

Variables	Range
Main channel diameter, D (mm)	1–3.2
Cyanex 272 concentration, (% *v*/*v*)	10–30
Flowrate ratio, Q_org_/Q_aq_	0.5 and 1
Residence time, t (s)	2–60
Reactor length (cm)	8.5–250
Mixture velocity, u_mix_ (m/s)	0.0029–0.3
Organic (aqueous) phase flowrate, Q_org_ (Q_aq_) (mL/min)	10–70
